# A Systematic Analysis of Eluted Fraction of Plasma Post Immunoaffinity Depletion: Implications in Biomarker Discovery

**DOI:** 10.1371/journal.pone.0024442

**Published:** 2011-09-07

**Authors:** Amit Kumar Yadav, Gourav Bhardwaj, Trayambak Basak, Dhirendra Kumar, Shadab Ahmad, Ruby Priyadarshini, Ashish Kumar Singh, Debasis Dash, Shantanu Sengupta

**Affiliations:** 1 G. N. Ramachandran Knowledge Center for Genome Informatics, Institute of Genomics and Integrative Biology (Council of Scientific and Industrial Research), Delhi, India; 2 Proteomics and Structural Biology Division, Institute of Genomics and Integrative Biology (Council of Scientific and Industrial Research), Delhi, India; University of Hyderabad, India

## Abstract

Plasma is the most easily accessible source for biomarker discovery in clinical proteomics. However, identifying potential biomarkers from plasma is a challenge given the large dynamic range of proteins. The potential biomarkers in plasma are generally present at very low abundance levels and hence identification of these low abundance proteins necessitates the depletion of highly abundant proteins. Sample pre-fractionation using immuno-depletion of high abundance proteins using multi-affinity removal system (MARS) has been a popular method to deplete multiple high abundance proteins. However, depletion of these abundant proteins can result in concomitant removal of low abundant proteins. Although there are some reports suggesting the removal of non-targeted proteins, the predominant view is that number of such proteins is small. In this study, we identified proteins that are removed along with the targeted high abundant proteins. Three plasma samples were depleted using each of the three MARS (Hu-6, Hu-14 and Proteoprep 20) cartridges. The affinity bound fractions were subjected to gelC-MS using an LTQ-Orbitrap instrument. Using four database search algorithms including MassWiz (developed in house), we selected the peptides identified at <1% FDR. Peptides identified by at least two algorithms were selected for protein identification. After this rigorous bioinformatics analysis, we identified 101 proteins with high confidence. Thus, we believe that for biomarker discovery and proper quantitation of proteins, it might be better to study both bound and depleted fractions from any MARS depleted plasma sample.

## Introduction

Proteomics is an important tool to identify relevant biomarkers for prognosis or diagnosis of various diseases. Plasma is the most preferred diagnostic material for disease proteomic studies due to its non-invasive nature. It is a heterogeneous collection of proteins secreted or leaked from all types of tissues revealing the cellular state due to spatio-temporal differences in protein expression. Thus, being a direct reflection of the patho-physiological condition of a patient, it is considered to be a diagnostic goldmine for biomarkers[1]. But, it is also one of the most difficult body fluids to work with because of the sample complexity and wide dynamic range of abundance spanning >12 orders of magnitude[2].

Many studies have emphasized the importance of plasma as a treasure-trove for biomarker discovery[3]. About 95% of the plasma proteome is accounted by only 10–12 highly abundant proteins; the remaining 5% being in extremely low abundance. However, it is this low abundance fraction of proteome that contains tissue leakage proteins and proteins derived from pathological sources containing information on the onset and progression of a disease[1]. Hence, accurate profiling of changes in protein expression patterns could give critical insights into the development of a potential biomarker for clinical diagnostics. It is a non-trivial task to identify and validate them due to the abundance complexity as they get masked by large and abundant proteins. A “divide and conquer” strategy works best in exploration and cataloguing the plasma proteins[2], [4], [5]. By depleting plasma of the high-abundance proteins, the sample complexity is reduced[6] and makes the identification of low-abundance proteins tenable[7]–[9]. Although there are various methods used for depletion of one or more of the abundant proteins in the plasma, immunoaffinity based method, which allows simultaneous depletion of multiple high abundant proteins is widely used[10], [11].

Sample pre-fractionation using multi affinity removal system (MARS) has been shown to improve detection of low abundance proteins in plasma[12]. This technique employs a combination of antibodies that can bind to the highly abundant proteins thereby depleting their total concentration from plasma resulting in the reduction of its dynamic range and enrichment of low abundance-protein species. However, one of the potential drawbacks of removal of abundant proteins (targeted proteins against which antibodies are present in MARS) from the plasma is the simultaneous removal of some less abundant proteins (non-targeted proteins). Although there have been studies addressing the questions of non-targeted removal of a few proteins and the non-removal of some targeted ones, it is predominantly reported that both these categories have negligible number of proteins[8], [13] and these effects are minimal. With the advent of high resolution mass spectrometers it is now possible to identify proteins which could not be identified earlier using instruments with lower resolution and sensitivity. Thus, we tried to understand the extent of removal of non-targeted proteins by MARS columns- Hu6, Hu14 and Proteoprep 20 that targets the removal of 6, 14 and 20 abundant proteins respectively. Using an LTQ-Orbitrap instrument, we intended to identify a high-confidence set of proteins from the bound fraction and to ascertain if some of these proteins are being specifically removed because of their interaction with targeted proteins. The significance of such a study cannot be overstated if we follow that most biomarkers are found in low abundance in plasma. Since most high abundance proteins act as carrier proteins and take away many other proteins with them during MARS removal, there is a loss of proteins that may turn out to be potential biomarkers.

Using multiple algorithms to identify high-confidence non-targeted proteins and interaction analyses of high-abundance proteins, we have identified 101 non-targeted proteins and shown that non-targeted removal of proteins is not trivial as portrayed earlier and attempted to observe sampling variations and MARS column specific removal. We speculate that some proteins may be specifically removed due to their interactions and propensity to bind to high abundant proteins. To carry out an effective biomarker discovery exercise, we believe that the MARS bound fractions should not be ignored. Therefore, an integrative analysis approach profiling the non-targeted proteins from the MARS bound fraction along with the depleted one may prove better for biomarker discovery pipelines.

## Results

### Plasma Depletion

**Figure 1:**
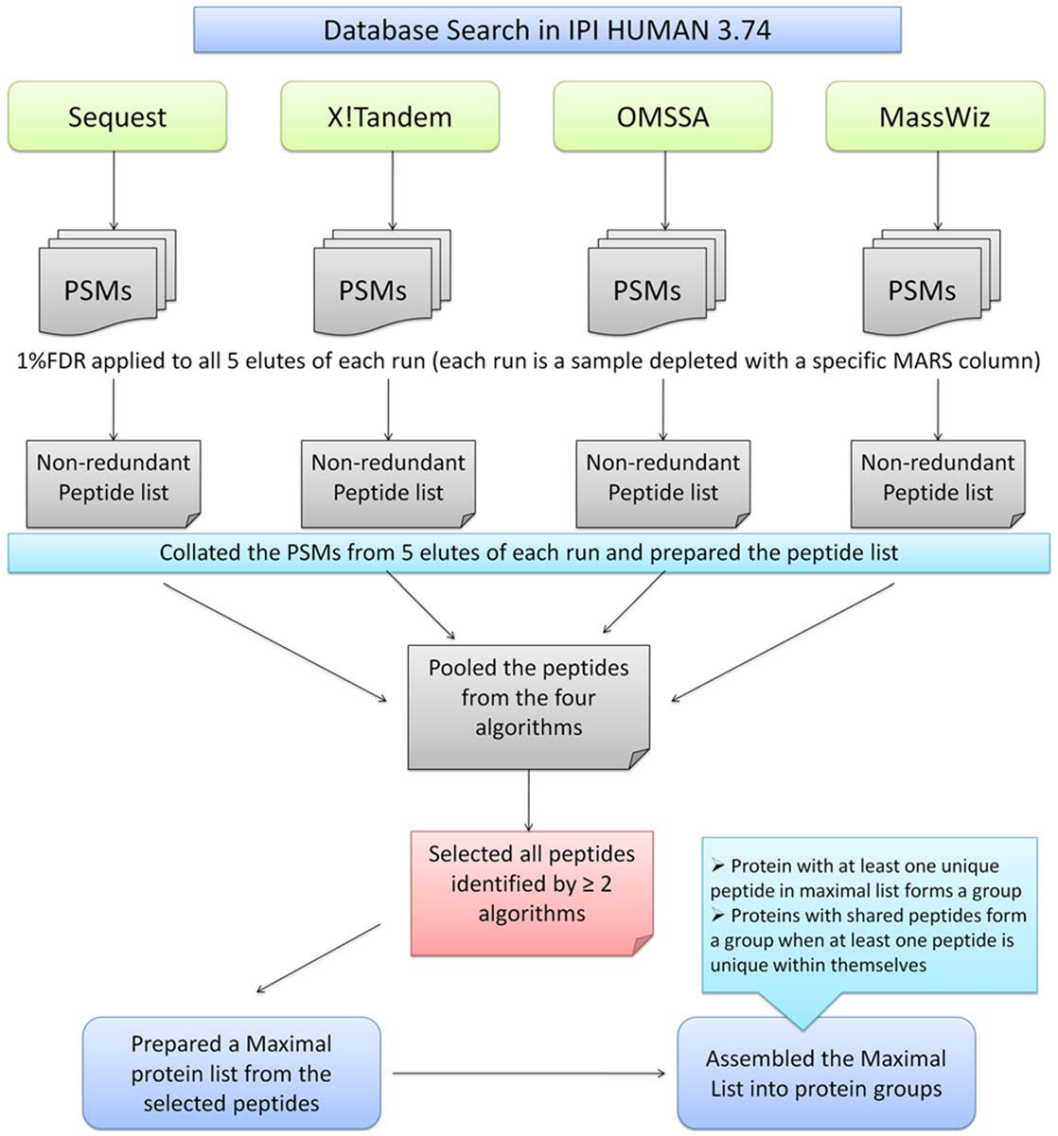
Overview of bioinformatics analysis workflow for mining the plasma peptides. Sequest, X!Tandem, OMSSA and MassWiz algorithms were used to identify PSMs at <1% FDR. All peptides identified by at least two algorithms were used for inferring the minimal protein list.

**Figure 2:**
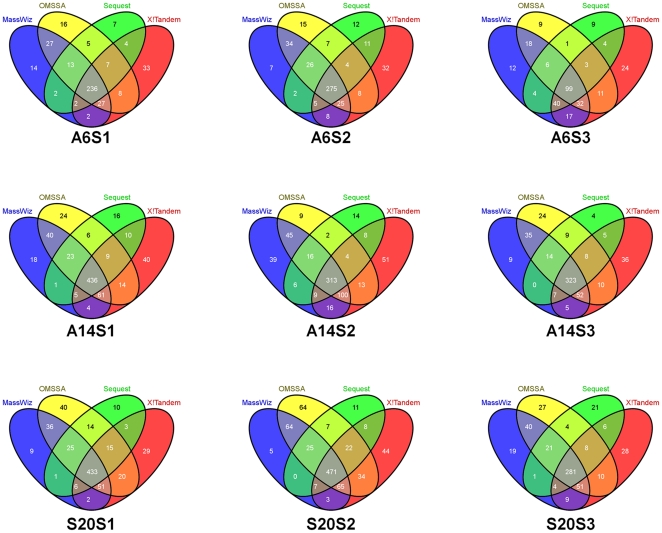
Venn diagram of peptides identified by different algorithms across samples and cartridges. A large portion of peptides cannot be picked by a single search algorithm and thus multiple algorithms can increase confidence as well as identify more peptides as shown.

Three human plasma samples were each depleted using three different MARS cartridges (A6, A14 and S20). The depletion efficiency of the three systems was above 90% for each of the sample. A comparative efficiency of protein enrichment is shown in Table 1. All the 9 eluted samples (3 samples with 3 cartridges) were subjected to GeLC-MS as mentioned in the methods. Since each lane of the gel was cut in 5 pieces, there were 45 fractions in total from the 9 eluted samples. The total number of peptides obtained from each fraction is shown in Table S1. However, since all these peptides are not of high confidence, an integrated bioinformatics workflow for high specificity peptide identification from plasma was developed (Figure 1). In this workflow, separate target-decoy searches were conducted in four different algorithms- Sequest, X!Tandem, OMSSA and MassWiz. A stringent FDR of <1% was applied. The objective of such an effort was to characterize plasma proteins with high confidence, keeping false positives to a bare minimum. Use of multiple algorithms increases the statistical confidence[14] if the same peptide is picked up by more than one search algorithm. The Venn diagrams in figure 2 illustrate the number of non-redundant peptides assigned by each algorithm and a high mutual agreement is evident in most cases. The total number of peptides (at<1%FDR) and selected high-confidence peptides (≥2 algorithms) are shown in figure S1. The total number of peptides identified by at least one, two, three and four algorithms is shown in Table S1. All peptides identified by a single algorithm were discarded so as to keep a high confidence set only. This prevents identification of an incorrect protein based on a single peptide hit. As shown by Gupta et al., removing single peptide hits leads to a substantial loss in true protein hits[15]. From this high-confidence set (identified by at least two algorithms), the peptides were used for protein inference where protein groups were formed as described in methods section. All targeted proteins (those which are supposed to be removed by MARS cartridge), keratins and immunoglobulins were removed from the protein groups. The remaining protein groups were reserved for analyses. From here on, we use the term “proteins” for “protein groups” that we have identified.

**Table 1:** Separation efficiency of cartridges is shown for MARS A6, A14 and S20.

Depletion System (supplier)	Average Plasma Protein load(µg)	Average of Total protein yield after depletion (µg)	Expected depletion efficiency (%)	Observed depletion efficiency (%)
A6	10 µl (720)	72.0	92–94	90
A14	10 µl (720)	61.2	94	91.5
S20	10 µl (720)	36.0	99	95

More than 90% efficiency was observed for all cartridges. All values given in the table are mean of three plasma samples.

### Analysis of bound fraction

**Figure 3:**
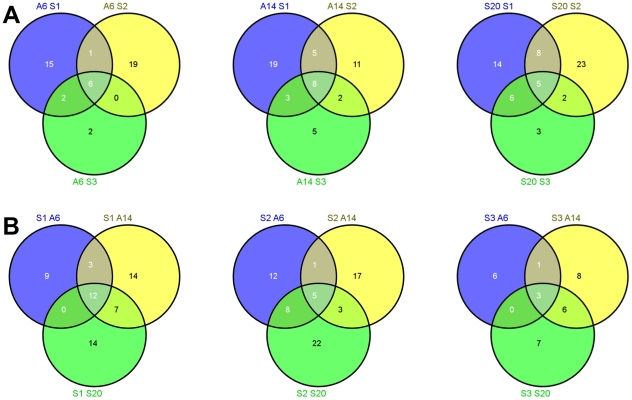
(A) Comparison of proteins (IPI identifiers) from a specific cartridge. (B) Comparison of proteins (IPI identifiers) from a specific sample across cartridges.

The number of proteins identified from each fraction of the depletion systems after excluding the target proteins of the cartridge in consideration, are shown in Table 2. Redundancy in the list of proteins was corrected manually. A total of 101 distinct plasma proteins were identified and their detailed information is listed in Table S2. Proteins identified from three plasma samples for a single cartridge were pooled and a non-redundant list was made for the three depletion systems. The number of proteins identified in different MARS is 45, 53 and 61 respectively for A6, A14 and S20 when the protein lists for all samples were combined (Figure S2) indicating that the total number of non-targeted proteins in the bound fraction increased with increase in the numbers of proteins that are targeted for removal. Further, 9, 18 and 21 proteins were found to be common in at least two samples eluted from A6, A14 and S20 respectively (Figure 3A) while 22, 17 and 10 proteins were eluted from at least 2 cartridges using a specific sample- S1, S2 and S3 respectively (Figure 3B).

**Table 2:** The numbers of unique non-targeted proteins identified in the eluted fractions excluding variants of immunoglobulin, targeted proteins and keratins from each sample after depletion with the three cartridges are shown here.

	MARS		
Sample	A6	A14	S20
**S1**	24	36	33
**S2**	26	26	38
**S3**	10	18	16

**Figure 4:**
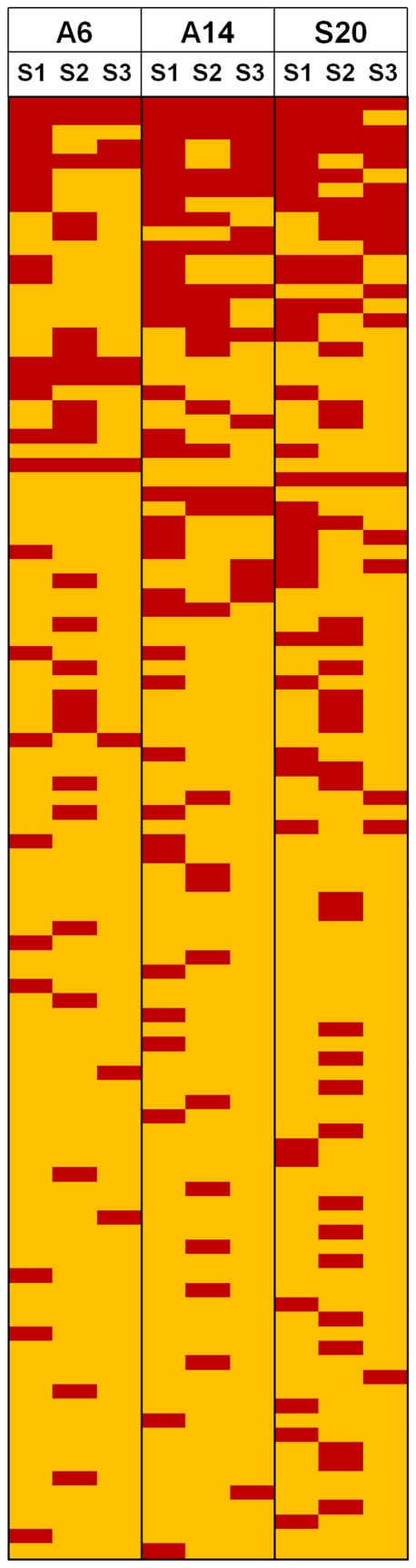
Heat map depicting concordance of proteins within and across cartridges.

The identified proteins were sub-categorized as “common proteins” and “unique proteins” based on the fact that if a protein falls in any two cells of the 3×3 matrix in Table 2, it is considered under “common proteins”. The rest (found in a single sample and a single MARS cartridge) were designated as “unique proteins”. The common proteins could be further classified as (A) Proteins common between at least two different samples (Table S2A) and (B) Proteins which are common in a particular sample but from different cartridges (Table S2B) and (C) proteins that were unique to a specific sample and cartridge (Table S2C). Thus, we identified 38 proteins that were eluted in at least 2 different samples (Group A), 14 proteins that were eluted from any single sample but in multiple cartridges (Group B) and 49 proteins that were unique to a specific sample and cartridge (Group C). The matrix of identified proteins with respect to the MARS column is depicted as a heat map for direct visual comparison (Figure 4). The heat map revealed that some proteins are constantly removed across samples, cartridges or both. For instance Dermcidin, Apolipoprotein D, Apolipoprotein E, Elongation factor 1-alpha. These proteins can be visualized in the upper part of the heat map. Of the 101 proteins that we identified in this study, 53 proteins (28 in group A, 9 in group B and 16 in group C) have been reported to be present in the eluted fraction in earlier studies using various depletion methods (Table S2). Thus, we are for the first time reporting the presence of an additional 48 proteins in the MARS bound fraction.

We also checked how many of the proteins that we found in the eluted fraction are listed in the Plasma Proteome Database (PPD)[16]. For this, the uniprot id and the corresponding Refseq/ Entrez ids of all 101 proteins were mined for the IPI accessions and searched in Human Protein Reference Database (HPRD)[17] and PPD. These periodically updated databases are a curated compendium of high quality human proteomics data which acts as a reference for the proteomics community [17]–[21]. We found that from our list of 101 proteins, 99 were present in HPRD and 95 were found in PPD depicting a high concordance with these databases. The specificity of non-targeted protein removal can be sample specific or MARS specific. Venn diagrams depicting the proteins identified from different samples for a particular MARS (Figure 3A) and different MARS for a particular sample (Figure 3B) can reveal such effects. Figure 3A shows that A6 had 6, A14 had 8 and S20 had 5 proteins common to all samples. This suggests that these proteins may be specific to the MARS used. Similarly, figure 3B suggests 12, 5 and 3 proteins to be sample specific for sample 1, sample 2 and sample 3 respectively since these were found in all MARS for the particular sample used.

**Figure 5:**
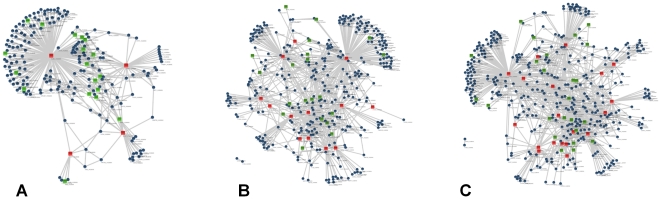
The interaction maps of (A) A6, (B) A14 and (C) S20 where the red squares denote targeted proteins; blue circles denote all interaction partners while the non-targeted proteins are represented with green squares.

One of the major reasons for the removal of non-targeted proteins might be their interaction with the targeted proteins. Thus, to check the proteins that interact with the targeted proteins we used Cytoscape which is a widely used network visualization and interaction analysis tool. Cytoscape was used for creating interaction networks for proteins that are targeted to be removed by each of the MARS cartridges. The interaction networks are shown in figure 5. These interacting partners were matched with our protein list to check the overlap and evaluate the specificity of the removal. 18, 20 and 24 non- targeted proteins were found to interact with one or more of the targeted proteins in MARS A6, A14 and S20 respectively. These proteins when combined make a non-redundant list of 33 proteins indicating that of the 101 proteins that we identified 33 of them are known to interact with the targeted abundant proteins.

Thus, our observations clearly indicate that non-targeted proteins in the bound fraction are much higher in number. Some of the proteins that we identified in the eluted fraction have earlier been reported to be markers for certain disease states. For instance- Retinol Binding Protein-4 has been reported to be a biomarker for renal dysfunction and cardiovascular disease in type 2 diabetes [22]. Vitronectin concentrations have been reported to predict the risk in patients undergoing coronary stenting[23]. S100A8 is identified as a biomarker of HPV-18 infected oral squamous cell carcinomas by Lo et al.[24]. Serum paraoxonase PON1 has been reported as a potential biomarker of organophosphate toxicity[25]. Nicholas et al. have reported Apo A1 and Lipocalin as biomarker for chronic obstructive pulmonary disease[26].

## Discussion

Plasma pre-fractionation has proved to be necessary since it reduces the sample complexity and helps enrich low abundance proteins by removing highly abundant proteins. However, it also creates a problem due to concomitant removal of non-targeted ones. Choosing a depletion system remains a trade-off between what we want to remove versus what gets lost. The plausible reasons for the removal of the non-targeted proteins may be either protein-protein or protein-antibody interactions. In this study, protein-antibody interactions were not studied in detail since these depletion systems have been proved to be sufficiently sensitive and specific [8], [13]. On the other hand interaction of targeted proteins with other proteins seems to be the most plausible reason for the removal of non-targeted proteins. For instance, albumin, a carrier protein, is known to be bound with different small molecular weight proteins [27], [28]. This sort of binding could be specific or non-specific. Gundry et.al.[28], have described the ‘albuminome’, which consists of 35 proteins from plasma that are co-eluted with albumin using anti HSA (human serum albumin) depletion system. These were shown to be potential candidates for non-targeted removal during albumin depletion. From our list of identified non-targeted proteins we found 27 proteins matching with the list of ‘albuminome’. So, it is likely that the removal of these proteins is due to the specific binding property of albumin with other proteins.

Stempfer et al.[29] reported 24 proteins from the bound fraction using MARS-6, of which 15 proteins matched with the proteins identified in this study. A detailed assessment of IgY-12 depletion systems by Liu et al.[30], and also by Huang et al.[31] pointed towards some non-targeted removal from depletion of plasma or serum. From their studies, 24 and 5 proteins respectively matched with our list. Similar efforts have been taken by Zhou et al.[32] by depleting 6 most abundant proteins from plasma. Gong et al.[33] have reported the removal of 129 non-targeted proteins, the largest number while depleting six most abundant proteins. However, in this the data analysis was carried out using only Sequest and may have inadvertently over-represented the number of proteins since single-peptide identifications from one algorithm are more prone to be false in comparison to those from multiple algorithms. For instance, if we had used a single algorithm (Sequest) to analyse our data we would have identified 112 proteins from A6 bound fraction, whereas using the stringent criteria of our study we identified 45 proteins from the A6 bound fraction (data not shown). Further, in our analysis we have removed all the immunoglobulin variants, keratin family of proteins and variants of the respective targeted proteins.

In contrast to these studies, Bellei et al.[13] have shown MARS-6 to be highly specific. Only 3 non-targeted proteins were identified in the bound fraction using nano LC-CHIP-MS analysis. The controversy regarding the extent of non-targeted protein removal remains a major issue in plasma proteomic studies. But it is important that the identified proteins should be unambiguous and highly-confident. Therefore stringency in bioinformatics analysis for the identification of proteins in bound fraction is the most important step. According to Tu et al.[8]; a total of 23 proteins were found to be present in bound fractions of MARS-7 and MARS-14, of which 9 proteins matched with our data. A broad literature search points towards the fact that the issue of non-targeted removal should be evaluated in detail.

Recently, Smith et al.[34] have analyzed the potential efficiency of the three depletion systems that we have used. Interestingly, in an attempt to identify the proteins from bound fraction using a ‘top-down’ approach they could not identify any non-targeted proteins in the bound fraction. The stringent criterion for the identification of proteins is important to understand non-targeted removal of proteins during depletion of plasma. Mining the plasma proteome has always been a major challenge. Interaction analysis of different abundant proteins suggests that some specific removal will always be there during depletion of different abundant proteins from plasma. There are several targeted proteins which may have a significant role in non-targeted removal. For eg, Histidine rich glycoprotein and Antithrombin interact with plasminogen, ATP Synthase subunit beta interacts with Plasminogen, Serum paraoxonase interacts with Apo-A1 etc. It is important to know the proteins which could be removed specifically.

While the evidence presented here is not enough to conclude that these non-targeted proteins will not be found in the depleted fraction of plasma but it will however definitely be an impediment in proper quantitation of such proteins. The distribution of the non-targeted proteins in the depleted and the bound fractions might be dictated by the binding affinity of the non-targeted proteins to the targeted proteins and the concentration of the non-targeted protein in a particular plasma sample. Thus, if the binding affinity of a particular non-targeted protein to the targeted protein is low it is likely that a part of it will be eluted along with the abundant targeted proteins while the rest will remain in the depleted fraction. This understanding will ultimately help in devising proper downstream analysis of depleted fraction from plasma in discovery based clinical proteomic studies which now rely heavily on the protein quantitation. However, it might be prudent not to consider such proteins as biomarkers unless it has been evaluated in large number of samples.

### Conclusion

Analysis of plasma samples for the discovery of new disease biomarkers holds great promise; but is fraught with challenges [3], [35], [36]. The potential of affinity based pre-fractionation of plasma can be maximized when the downstream workflow utilizes both the fractions (flow-through and bound fraction) for analysis. We have shown that the bound fractions contain important plasma proteins which have been indicated to be relevant as disease biomarkers. Contrary to general belief, our study reports more proteins in non-targeted category. Using this approach for simplifying the plasma proteome is more beneficial when both the fractions are analyzed. The effect of MARS on enrichment of sample is the next question to address and thus forms the immediate focus of our next work. Further study on this aspect would provide new insights after in-depth analysis of the depleted fractions.

## Materials and Methods

### Sample Collection and Plasma Isolation

Blood samples were collected in EDTA vacutainer from three healthy individuals. Ethics Committee f the Institute of Genomics and Integrative Biology (Institutional human ethics committee, IHEC) approved the collection of human blood samples. Written consent from all the individuals were obtained on the day of recruitment. The blood samples were stored upright at 4°C until they were spun at 2500 rpm at 4°C for 15 minutes as mentioned in Omenn et al [37]. The separated plasma was aliquoted and stored at −80°C for further analysis.

### Depletion of Plasma Samples

The depletion was performed according to the manufacturers' protocol for all the three depletion cartridges. The list of targeted proteins for each cartridge is given below. From here on, we use the terms A6 for Hu-6, A14 for Hu-14 and S20 for Proteoprep 20 cartridges.

A6:- Albumin, IgG, Antitrypsin, IgA, Transferrin, Heptoglobin.

A14:- Albumin, IgG, Antitrypsin, IgA, Transferrin, Haptoglobin, Fibrinogen, Alpha 2-macroglobulin, Alpha 1-macroglobulin, IgM, Apolipoprotein A1, Apolipoprotein A2, Complement C3, Transthyretin.

S20:- Albumin, IgG, Transferrin, Fibrinogen, IgA, Alpha 2-macroglobulin, IgMs, Alpha1- Antitrypsin, Complement C3, Haptoglobin, ApoA1, ApoA2, Apo B, Alpha 1 Glycoprotein, Ceruloplasmin, Complement C4, Complement A1q, IgD, Prealbumin, Plasminogen.

### Sample Preparation and SDS-PAGE

The proteins in the bound fraction were concentrated from a volume of 4 ml to about 300 µl using centrifugal concentrators (Millipore 3kd). The samples were precipitated with 2D clean up kit (Amersham) prior to SDS-PAGE. The protein pellets were resuspended in 7 M urea, 2 M thiourea, 25 mM ammonium bicarbonate buffer (pH 8.5). 30 µg of protein from each of the three samples eluted using three cartridges was subjected to gel electrophoresis on a 10% tricine gel and stained with phast gel coomassie (Amersham).

### In-Gel Digestion

Each lane of eluted fractions in the gel was excised and sliced into 5 fractions. Each fraction was washed with LC-MS grade water (Sigma) 2–3 times and destained at least twice with the destaining solution [100%MeOH:50 mM Ammonium bicarbonate (1∶1)]. The gel pieces were then dehydrated in 100% ACN:50 mM Ammonium bicarbonate (1∶1) and reduced using freshly prepared DTT (25 mM) for 20 minutes (55°C) and alkylated using IAA (55 mM) for 30 minutes in dark at room temperature. The samples were then dried using speed vac (Eppendorf). Trypsin (promega V511A, 12.5 ng/µl) was added to these samples and incubated for 30 minutes on ice until the gel pieces swelled and then overlaid with 10 mM Ammonium bicarbonate buffer. The tubes were left overnight at 37°C and extraction of the tryptic peptides was carried out with 1% TFA in 30% ACN.

### Nano-RP-LC MS/MS analysis

Lyophilized peptides were reconstituted with 2% ACN supplemented with 0.1% formic acid. The peptides were subjected to reverse phase chromatographic fractionation through split free nano-LC system (EASY-nLC; Proxeon Biosystems now Thermo Fisher Scientific) coupled to the LTQ Orbitrap mass spectrometer (LTQ-Orbitrap XL, Thermo Fisher Scientific).

Chromatography was performed using two buffer systems, viz; Buffer A- 2% ACN, 0.1% formic acid and Buffer B-98% ACN and 0.1% formic acid. The pre-column (fused silica (f.s.) capillary of 100 µm internal diameter, length 3 cm of Synergy reversed phase C-18 beads) was equilibrated with 30 µl of buffer A at a flow rate of 6 µl/min. The f.s. capillary of internal diameter 100 µm and length 10 cm was pulled to make the nanospray tip/needle for electrospray ionization. Synergy reversed phase C-18 beads were then packed on to this nanospray needle and thus the analytical column was ready, which was equilibrated with 10 µl of buffer A at a flow rate of 0.8 µl/min. Samples were then loaded onto the pre-column for desalting and then to the analytical column. The peptides were separated using a 140 min stepwise gradient of 15% Buffer B for 20 min, 45% for 110 min and 100% for 2–8 min at a constant flow rate of 300 nl/min. The LTQ-Orbitrap XL mass spectrometer was operated in data dependent MS/MS mode with a MS survey scan (m/z 350 -2000) at resolution set to 60,000 in FTMS mode followed by four data-dependent scans in ITMS mode in which the four most intense ions were successively subjected to CID (MS/MS). Dynamic mass exclusion was enabled with a repeat count of once every 30 seconds for a list size of 500.

### Database Searching and Statistical analysis

The RAW files obtained from 45 LC-MS/MS runs (5 gel slices×3 samples×3 MARS) were converted to mascot generic format (mgf) using msconvert.exe program from ProteoWizard[38] version 1.6. These mgf files were then independently searched with four different database search algorithms for decreasing false positives in peptide identifications. The overview of search and statistical analyses performed is summarized in figure 1. We have used four algorithms namely- Sequest[39] version 28.0.0.0, OMSSA[40] version 2.1.9, X!Tandem[41] version Tornado and our in-house developed algorithm MassWiz[42] version 1.7.0.0 to search against Human IPI database[43] (database v.3.74). Since the spectra acquisition was carried out in an LTQ-Orbitrap XL instrument with LTQ for fragmentation, the database searches were performed with 10 ppm precursor and 0.6 Da fragment ion tolerances. All cysteines were considered modified with carbamidomethylation (+57.012) and a variable modification of methionine oxidation (+15.9949) was also taken into account. Tryptic digestion with a maximum of 2 missed cleavages was considered. An FDR threshold of <1% was chosen for keeping false positives to a minimum. A separate target-decoy search strategy[44] was used for FDR estimation.

Sequest searches and FDR calculations were conducted using Proteome Discoverer 1.1 and PSMs (Peptide Spectrum Matches) from different runs were merged. OMSSA searches were queued in a batch mode using a perl program and results were saved in .csv format. The FDR was calculated on e-values using another perl program. The results from different runs were merged together. X!Tandem was also run through a Perl program in batch mode. Another Perl program was used to parse XML results and FDR was calculated based on e-values. The results from different runs were then merged.

The in-house developed algorithm MassWiz was also used to analyze the data. MassWiz was developed with a view to maximize the use of information content from the MS/MS fragment spectra in a high-throughput fashion. Target-Decoy strategy is inherently integrated into MassWiz search. All searches were conducted using the aforementioned parameters. After database searches, the different runs were merged as described earlier for other algorithms.

For all datasets, peptides identified by at least two algorithms were selected for further analysis. This further increased confidence and decreased false positives. By applying this stringent criterion, the actual FDR is expected to be much lower than the original FDR threshold of 1% applied earlier. Based on these peptides, protein grouping was performed following the principles of peptide parsimony as described by Nesvizhski and Aebersold[45]. Briefly, the program groups the proteins where the protein group representative must have either one unique peptide or the group must have at least one unique peptide exclusively to its members. For compiling a final list of proteins, only the selected peptides (≥2 algorithms) were mapped back to the proteins for sequence coverage calculation. The peptides which had ambiguity in group assignment (bridging multiple groups) were discarded. In case of isoforms, if a particular isoform is identified by unique peptide(s), it was designated as group representative. In cases where all isoforms shared the peptide(s), the isoform with longest protein length was designated as the group representative. The proteins identified by a single peptide were manually validated as per MIAPE guidelines[46]. The spectral images for validation were generated using the pLabel tool in the pFind[47] suite. The manual validation file is provided as Spreadsheet S1 and annotated images are provided as Archive S1.

### Protein Interaction Network Analysis

To create the interaction network of the targeted proteins, we used Cytoscape[48] version 2.8. Uniprot identifiers of each target protein were provided as input to the APID2NET[49] plugin to build the network. Wherever there were multiple isoforms of the target proteins, all were taken to build the interaction network. The interaction networks were separately created for different MARS.

### Online data submission

The data associated with this manuscript may be downloaded from ProteomeCommons.org Tranche using the following hash:

GHBbTxIbroyf1JPdTECnsFFChIcHNnIjLZzdWtKte8j892JBsRi3KF2/RdFvZmLspptDv4S/HJgyc+zC8hoDeoPP8J4AAAAAAAACow =  = 

The hash may be used to prove exactly what files were published as part of this manuscript's data set, and the hash may also be used to check that the data has not changed since publication.

## Supporting Information

Figure S1 **Total peptides pooled after 1%FDR from four algorithms-Sequest, X!Tandem, OMSSA and MassWiz.** The peptides identified by at least 2 algorithms were selected for further analysis.
(TIF)

Figure S2 **Number of proteins identified in the bound fraction from the three removal systems.**
(TIF)

Table S1 **Number of peptides identified by at least 1, 2, 3 and 4 algorithms.** The four algorithms used were MassWiz, SEQUEST, X!Tandem and OMSSA. The peptides identified by ≥2 algorithms were selected for further analyses.
(DOC)

Table S2 **List of all non-targeted proteins from the bound fractions categorized as- (A) Proteins Common between Samples, (B) Proteins Common in a particular sample across different cartridge and (C) Proteins Unique to a sample and cartridge.**
(DOC)

Spreadsheet S1 **List of manually validated peptides for single peptide hit proteins, their raw spectral counts and algorithms that identified the peptides.**
(XLSX)

Archive S1 **Annotated spectral images for the single peptide hits.**
(RAR)
