# Supplementary figures and images for: A Systematic Analysis of Eluted Fraction of Plasma Post Immunoaffinity Depletion: Implications in Biomarker Discovery
Source: PLoS One. 2011 Sep 7;6(9):e24442. doi: 10.1371/journal.pone.0024442 (PMC3168506; doi:10.1371/journal.pone.0024442)

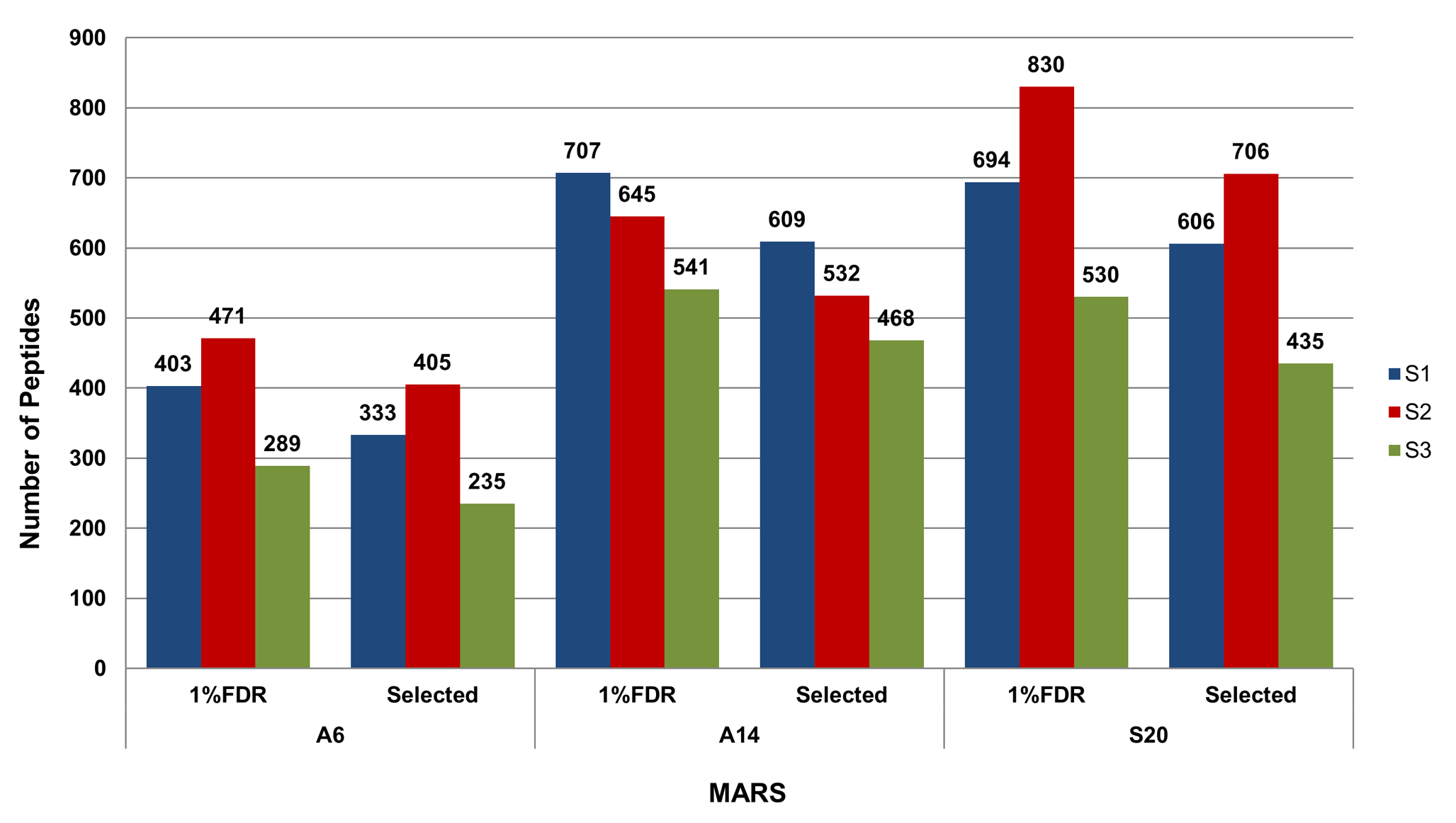

Supplement: Figure S1 — Total peptides pooled after 1%FDR from four algorithms-Sequest, X!Tandem, OMSSA and MassWiz. The peptides identified by at least 2 algorithms were selected for further analysis. (TIF) [file pone.0024442.s001.tif]

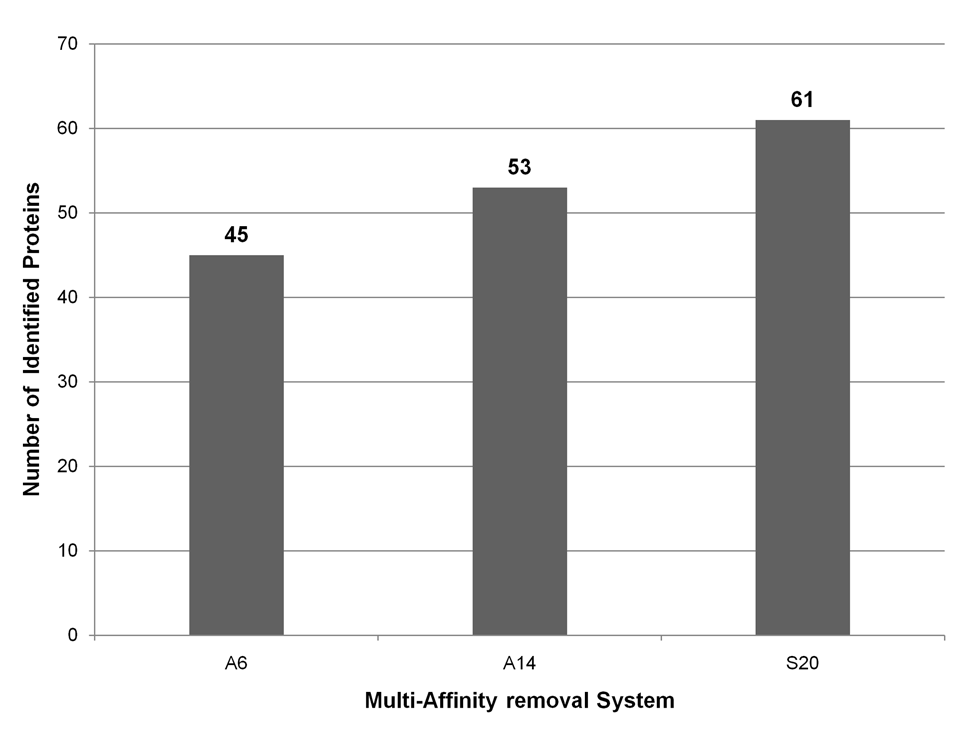

Supplement: Figure S2 — Number of proteins identified in the bound fraction from the three removal systems. (TIF) [file pone.0024442.s002.tif]
