# Supplementary material for: A Systematic Analysis of Eluted Fraction of Plasma Post Immunoaffinity Depletion: Implications in Biomarker Discovery
Source: PLoS One. 2011 Sep 7;6(9):e24442. doi: 10.1371/journal.pone.0024442 (PMC3168506; doi:10.1371/journal.pone.0024442)
Supplement: Table S1 — Number of peptides identified by at least 1, 2, 3 and 4 algorithms. The four algorithms used were MassWiz, SEQUEST, X!Tandem and OMSSA. The peptides identified by ≥2 algorithms were selected for further analyses. (DOC) [file pone.0024442.s003.doc]

Supplementary Table 1: Number of peptides identified by at least 1, 2, 3 and 4 algorithms. The four algorithms used were MassWiz, SEQUEST, X!Tandem and OMSSA.

| Sample and Cartridge | Peptides from any 1 or more algorithm | Peptides from any 2 or more algorithms | Peptides from any 3 or more algorithms | Peptides from at least 4 algorithms |
| --- | --- | --- | --- | --- |
| A6S1 | 403 | 333 | 285 | 236 |
| A6S2 | 471 | 405 | 335 | 275 |
| A6S3 | 289 | 235 | 180 | 99 |
| A14S1 | 707 | 609 | 534 | 435 |
| A14S2 | 645 | 532 | 442 | 313 |
| A14S3 | 541 | 468 | 404 | 323 |
| S20S1 | 694 | 606 | 530 | 433 |
| S20S2 | 830 | 706 | 590 | 471 |
| S20S3 | 530 | 435 | 365 | 283 |
