# Supplementary material for: A Systematic Analysis of Eluted Fraction of Plasma Post Immunoaffinity Depletion: Implications in Biomarker Discovery
Source: PLoS One. 2011 Sep 7;6(9):e24442. doi: 10.1371/journal.pone.0024442 (PMC3168506; doi:10.1371/journal.pone.0024442)
Supplement: Table S2 — List of all non-targeted proteins from the bound fractions categorized as- (A) Proteins Common between Samples, (B) Proteins Common in a particular sample across different cartridge and (C) Proteins Unique to a sample and cartridge. (DOC) [file pone.0024442.s004.doc]

Supplementary Table 2: List of all non-targeted proteins from the bound fractions

2A. Proteins Common between Samples

| **IPI ID** | **Description** | **Total Peptides** | **uniprot id** | **Interaction with targeted proteins^a^** | **Refseq/Entrez** | **Sequence Coverage (%)** | **Literature reports^b^** |
| --- | --- | --- | --- | --- | --- | --- | --- |
|
| IPI00032220.3 | AGT Angiotensinogen | 1 | P01019 | No | NP_000020/183 | 2.47 | [8,28,30,33] |
| IPI00186903.4 | APOL1 Isoform 2 of Apolipoprotein L1 | 2 | O14791-2 | Yes | NP_663318/8542 | 6.28 | [30–32] |
| IPI00030739.1 | APOM Apolipoprotein M | 2 | O95445 | No | NP_061974/55937 | 15.96 | [33] |
| IPI00289838.5 | ESCO1 Isoform 3 of N-acetyltransferase ESCO1 | 1 | Q5FWF5-3 | No | 114799 | 6.98 | - |
| IPI00339223.2 | FN1 Isoform 3 of Fibronectin | 1 | P02751-3 | No | NP_002017/2335 | 1.49 | [29,30,32] |
| IPI00645038.1 | ITIH2 Inter-alpha (Globulin) inhibitor H2 | 2 | Q5T985 | No | 3698 | 3.96 | - |
| IPI00936661.1 | LOC100291190 hypothetical protein XP_002347529 | 3 | - | No | - | 22.28 | - |
| IPI00847635.1 | SERPINA3 Isoform 1 of Alpha-1-antichymotrypsin | 3 | P01011-1 | No | NP_001076/12 | 9.22 | [8,13,28,33] |
| IPI00940393.1 | Elongation factor 1-alpha | 1 | Q61Q15 | No | 1915 | 2.78 | - |
| IPI00478003.2 | A2M Alpha-2-macroglobulin | 4 | P01023 | Yes | NP_000005 | 55.29 | [8,13,29,33] |
| IPI00021841.1 | APOA1 Apolipoprotein A-I | 10 | P02647 | Yes | NP_000030 | 75.28 | [8,13,28,29,32,33] |
| IPI00021854.1 | APOA2 Apolipoprotein A-II | 1 | P02652 | Yes | NP_001634 | 21 | [28,29,32] |
| IPI00006662.1 | APOD Apolipoprotein D | 5 | P05090 | Yes | NP_001638 | 29.63 | [30,31,33] |
| IPI00021842.1 | APOE Apolipoprotein E | 5 | P02649 | Yes | NP_000032 | 20.5 | [28,30,32] |
| IPI00166729.4 | AZGP1 Zinc-alpha-2-glycoprotein | 3 | P25311 | No | NP_001176 | 13.09 | [28,30,31,  33] |
| IPI00783987.2 | C3 Complement C3 (Fragment) | 12 | P01024 | Yes | NP_000055 | 61.4 | [13,30–32] |
| IPI00843913.3 | C4B Complement component 4A | 6 | B0V2C8,B2RUT6,Q6XMI6 | Yes | 720,Entrez | 21.67 | [8,13,28,29,32] |
| IPI00925214.1 | CASP12 Putative uncharacterized protein CASP12 | 1 | D6RJG1 | No | 120329 | 7.58 | - |
| IPI00025204.1 | CD5L CD5 antigen-like | 3 | O43866 | No | NP_005885 | 11.24 | [30,33] |
| IPI00795633.1 | CLU CLU | 5 | Q6LDQ3,Q8IWL5,Q8IWM0 | No | 1191 | 18.3 | [28,30,32] |
| IPI00644441.1 | CREB3L4 cAMP responsive element binding protein 3-like 4 | 1 | Q5T4L1 | No | 148327 | 8.33 | - |
| IPI00027547.2 | DCD Dermcidin | 4 | P81605 | Yes | NP_444513 | 35.45 | [29,32] |
| IPI00025753.2 | DSG1 Desmoglein-1 | 6 | Q02413 | No | NP_001933 | 11.25 | [29] |
| IPI00019568.1 | F2 Prothrombin (Fragment) | 3 | P00734 | Yes | NP_000497 | 8.04 | [28,32] |
| IPI00555812.5 | GC vitamin D-binding protein precursor | 2 | P02774-2 | No | NP_000574 | 12.87 | [28,30] |
| IPI00410714.5 | HBA1;HBA2 Hemoglobin subunit alpha | 5 | P69905 | Yes | NP_000508,NP_000549 | 63.38 | [8,28,32] |
| IPI00654755.3 | HBB Hemoglobin subunit beta | 10 | P68871 | Yes | NP_000509 | 82.99 | [8,28,30,32] |
| IPI00453473.6 | HIST2H4B histone cluster 2, H4b | 4 | P62805 | No | NP_001029249,NP_003486 | 42.72 | - |
| IPI00339274.5 | HIST2H2AC Histone H2A type 2-C | 2 | Q16777 | No | NP_003508 | 21.71 | - |
| IPI00022488.1 | HPX Hemopexin | 6 | P02790 | Yes | NP_000604 | 25.76 | [8,13,28,30,32,33] |
| IPI00022371.1 | HRG Histidine-rich glycoprotein | 5 | P04196 | Yes | NP_000403 | 12.19 | [28,30,33] |
| IPI00398625.5 | HRNR Hornerin | 12 | Q86YZ3 | No | NP_001009931 | 8.6 | [28] |
| IPI00916229.1 | MAT2A S-adenosylmethionine synthetase | 1 | B4DN45 | No | 4144 | 5.02 | - |
| IPI00845229.1 | PREX2 Isoform 2 of Phosphatidylinositol 3;4;5-trisphosphate-dependent Rac exchanger 2 protein | 1 | Q70Z35-2 | No | 80243 | 1.33 | - |
| IPI00019399.2 | SAA4 Serum amyloid A-4 protein | 2 | P35542 | No | NP_006503 | 15.38 | [13,28,30, 32] |
| IPI00032179.3 | SERPINC1 Antithrombin-III | 6 | P01008 | Yes | NP_000479 | 16.81 | [8,13,28,33] |
| IPI00291866.5 | SERPING1 Plasma protease C1 inhibitor | 7 | P05155 | Yes | NP_000053,NP_001027466 | 18.6 | [8,33] |
| IPI00298971.1 | VTN Vitronectin | 6 | P04004 | Yes | NP_000629 | 16.53 | [28–30,33] |

2B. Proteins Common in a particular sample across different cartridge

| **IPI ID** | **Description** | **Total Peptides** | **uniprot id** | **Interaction with targeted proteins^a^** | **Refseq/Entrez** | **Sequence Coverage (%)** | **Literature reports^b^** |
| --- | --- | --- | --- | --- | --- | --- | --- |
|
| IPI00304273.2 | APOA4 Apolipoprotein A-IV | 2 | P06727 | Yes | 337 | 6.06 | [13,28,32, 33] |
| IPI00013933.2 | DSP Isoform DPI of Desmoplakin | 12 | P15924-1 | No | NP_004406/1832 | 8.12 | [29] |
| IPI00873899.1 | ABCF1 Isoform 1 of ATP-binding cassette sub-family F member 1 | 1 | Q8NE71-1 | No | NP_001020262 | 1.3 | - |
| IPI00021857.1 | APOC3 Apolipoprotein C-III | 1 | P02656 | Yes | NP_000031 | 16.16 | [8,28,30,32] |
| IPI00398768.1 | ARG1 Isoform 2 of Arginase-1 | 2 | P05089-2 | No | 383 | 9.7 | - |
| IPI00021727.1 | C4BPA C4b-binding protein alpha chain | 2 | P04003 | Yes | NP_000706 | 4.36 | [33] |
| IPI00029739.5 | CFH Isoform 1 of Complement factor H | 1 | P08603-1 | Yes | NP_000177 | 1.46 | [30,32] |
| IPI00397801.4 | FLG2 Filaggrin-2 | 3 | Q5D862 | No | NP_001014364 | 1.76 | - |
| IPI00473011.3 | HBD Hemoglobin subunit delta | 8 | P02042 | No | NP_000510 | 63.95 | [31] |
| IPI00554711.3 | JUP Junction plakoglobin | 8 | P14923 | No | NP_002221 | 18.93 | [29] |
| IPI00019038.1 | LYZ Lysozyme C | 1 | P61626 | No | NP_000230 | 8.11 | [29] |
| IPI00022974.1 | PIP Prolactin-inducible protein | 2 | P12273 | No | NP_002643 | 17.81 | [29] |
| IPI00939362.1 | S100A9 Putative uncharacterized protein S100A9 | 2 | C9J1H1 | No | 6280 | 35.44 | - |
| IPI00307466.2 | SERPINB3 Isoform 2 of Serpin B3 | 1 | P29508-2 | No | 6317 | 7.1 | - |

2C. Proteins Unique to a sample and cartridge

| **IPI ID** | **Description** | **Total Peptides** | **uniprot id** | **Interaction with targeted proteins^a^** | **Refseq/Entrez** | **Sequence Coverage (%)** | **Literature reports^b^** |
| --- | --- | --- | --- | --- | --- | --- | --- |
|
| IPI00019580.1 | PLG Plasminogen | 2 | P00747 | Yes | NP_000292 | 23.46 | [28,30,32] |
| IPI00001310.8 | 18 kDa protein | 1 | - | No | - | 9.68 | - |
| IPI00022895.7 | A1BG Alpha-1B-glycoprotein | 2 | P04217-1 | No | NP_570602/1 | 5.05 | [13,28] |
| IPI00022426.1 | AMBP Protein AMBP | 3 | P02760 | Yes | NP_001624/259 | 13.64 | [30,33] |
| IPI00797556.1 | ANXA2 24 kDa protein | 2 | - | No | 302 | 12.33 | - |
| IPI00013885.1 | CASP14 Caspase-14 | 1 | P31944 | No | NP_036246/23581 | 4.55 | - |
| IPI00218390.1 | CD22 Isoform CD22-alpha of B-cell receptor CD22 | 1 | P20273-2 | No | 933 | 1.79 | - |
| IPI00032325.1 | CSTA Cystatin-A | 1 | P01040 | No | NP_005204/1475 | 12.24 | - |
| IPI00185361.6 | DDX55 ATP-dependent RNA helicase DDX55 | 1 | Q8NHQ9 | No | NP_065987/57696 | 1.17 | - |
| IPI00219713.1 | FGG Isoform Gamma-A of Fibrinogen gamma chain | 1 | P02679-2 | No | NP_000500/2266 | 56.75 | [33] |
| IPI00026256.2 | FLG Filaggrin | 1 | P20930 | No | NP_002007/2312 | 0.3 | - |
| IPI00219018.7 | GAPDH Glyceraldehyde-3-phosphate dehydrogenase | 1 | P04406 | No | NP_002037/2597 | 4.48 | - |
| IPI00020101.9 | HIST1H2BE;HIST1H2BC;HIST1H2BI;HIST1H2BG;HIST1H2BF Histone H2B type 1-C/E/F/G/I | 1 | P62807 | No | NP_003509/8347 | 11.9 | - |
| IPI00784154.1 | HSPD1 60 kDa heat shock protein; mitochondrial | 1 | P10809 | No | NP_002147/61054 | 3.84 | - |
| IPI00789324.3 | JUP cDNA FLJ60424; highly similar to Junction plakoglobin | 5 | B4DE59 | No | 3728 | 15.81 | - |
| IPI00654888.4 | KLKB1 Plasma kallikrein | 1 | P03952 | No | 3818 | 1.72 | - |
| IPI00009650.1 | LCN1 Lipocalin-1 | 3 | P31025 | No | NP_002288/3933 | 15.34 | - |
| IPI00152871.1 | LRRC15 Leucine-rich repeat-containing protein 15 | 1 | Q8TF66 | No | NP_570843/131578 | 1.72 | - |
| IPI00025094.3 | MYH16 cDNA: FLJ22037 fis; clone HEP08868 (Fragment) | 1 | Q9H6N6 | No | 84176 | 1.47 | - |
| IPI00288940.6 | OBSCN Isoform 1 of Obscurin | 1 | Q5VST9-1 | No | NP_001092093/84033 | 0.08 | - |
| IPI00942458.1 | RBM26 Isoform 1 of RNA-binding protein 26 | 1 | Q5T8P6-1 | No | 64062 | 1.49 | - |
| IPI00022420.3 | RBP4 Retinol-binding protein 4 | 1 | P02753 | Yes | NP_006735/23010 | 5.47 | [28] |
| IPI00879231.1 | SERPINF2 Alpha-2-antiplasmin | 1 | P08697 | Yes | NP_000925 | 2.24 | [30] |
| IPI00013890.2 | SFN Isoform 1 of 14-3-3 protein sigma | 2 | P31947-1 | No | NP_006133/2810 | 8.06 | [29] |
| IPI00300376.5 | TGM3 Protein-glutamine gamma-glutamyltransferase E | 2 | Q08188 | No | NP_003236/7053 | 3.61 | - |
| IPI00164776.2 | TMEM198 Transmembrane protein 198 | 1 | Q66K66 | No | NP_001005209/130612 | 2.5 | - |
| IPI00021440.1 | ACTG1 Actin; cytoplasmic 2 | 5 | P63261 | Yes | NP_001605 | 17.33 | [29] |
| IPI00218914.5 | ALDH1A1 Retinal dehydrogenase 1 | 1 | P00352 | No | NP_000680 | 2.59 | - |
| IPI00022229.2 | APOB Apolipoprotein B-100 | 8 | P04114 | Yes | NP_000375 | 9.69 | [8,30,33] |
| IPI00021855.1 | APOC1 Apolipoprotein C-I | 1 | P02654 | Yes | NP_001636 | 13.25 | [28,30,32,33] |
| IPI00298828.3 | APOH Beta-2-glycoprotein 1 | 1 | P02749 | Yes | NP_000033 | 4.93 | - |
| IPI00303476.1 | ATP5B ATP synthase subunit beta; mitochondrial | 2 | P06576 | Yes | NP_001677 | 9.26 | - |
| IPI00021536.2 | CALML5 Calmodulin-like protein 5 | 1 | Q9NZT1 | No | NP_059118 | 15.75 | - |
| IPI00892693.1 | CDSN Corneodesmosin | 1 | Q2L6G8 | No | 1041 | 4.35 | - |
| IPI00021033.2 | COL3A1 Isoform 1 of Collagen alpha-1(III) chain | 1 | P02461-1 | No | NP_000081 | 0.75 | - |
| IPI00942197.2 | DMKN 40 kDa protein | 1 | - | No | 93099 | 4.09 | - |
| IPI00029717.1 | FGA Isoform 2 of Fibrinogen alpha chain | 2 | P02671 | Yes | NP_068657 | 38.51 | [28,32] |
| IPI00298497.3 | FGB Fibrinogen beta chain | 1 | P02675 | Yes | NP_005132 | 67.82 | [33] |
| IPI00915753.1 | GULP1 Putative uncharacterized protein GULP1 | 1 | B8ZZ72 | No | 51454 | 4.12 | - |
| IPI00292530.1 | ITIH1 Inter-alpha-trypsin inhibitor heavy chain H1 | 1 | P19827 | Yes | NP_002206 | 1.32 | - |
| IPI00022417.4 | LRG1 Leucine-rich alpha-2-glycoprotein | 2 | P02750 | No | NP_443204 | 4.9 | [28,30,33] |
| IPI00022429.3 | ORM1 Alpha-1-acid glycoprotein 1 | 1 | P02763 | Yes | 5004 | 40.8 | [8,28,33] |
| IPI00027252.6 | PHB2 Prohibitin-2 | 1 | Q99623 | No | NP_001138303 | 4.01 | - |
| IPI00218732.3 | PON1 Serum paraoxonase/arylesterase 1 | 2 | P27169 | Yes | NP_000437 | 8.45 | [8,13,30, 33] |
| IPI00946754.1 | PRSS1 Protease serine 1 | 1 | Q45KI0,Q53ZX7,Q53ZX8 | No | 5644 | 9.15 | - |
| IPI00219806.7 | S100A7 Protein S100-A7 | 1 | P31151 | No | NP_002954 | 10.89 | [29] |
| IPI00007047.1 | S100A8 Protein S100-A8 | 3 | P05109 | No | NP_002955 | 32.26 | - |
| IPI00947285.1 | SBSN suprabasin isoform 1 precursor | 4 | Q6UWP8 | No | NP_001159506/374897 | 11.53 | - |
| IPI00797400.1 | UBB;RPS27A;UBC 19 kDa protein | 1 | P0CG47 | No | 7316 | 90.58 | - |

a) Found in at least any one MARS

b) These references discuss plasma depletion and affinity removal strategies
